# RNA components of the spliceosome regulate tissue- and cancer-specific alternative splicing

**DOI:** 10.1101/gr.246678.118

**Published:** 2019-10

**Authors:** Heidi Dvinge, Jamie Guenthoer, Peggy L. Porter, Robert K. Bradley

**Affiliations:** 1 Computational Biology Program, Public Health Sciences Division, Fred Hutchinson Cancer Research Center, Seattle, Washington 98109, USA;; 2 Basic Sciences Division, Fred Hutchinson Cancer Research Center, Seattle, Washington 98109, USA;; 3 Human Biology Division, Fred Hutchinson Cancer Research Center, Seattle, Washington 98109, USA

## Abstract

Alternative splicing of pre-mRNAs plays a pivotal role during the establishment and maintenance of human cell types. Characterizing the *trans*-acting regulatory proteins that control alternative splicing has therefore been the focus of much research. Recent work has established that even core protein components of the spliceosome, which are required for splicing to proceed, can nonetheless contribute to splicing regulation by modulating splice site choice. We here show that the RNA components of the spliceosome likewise influence alternative splicing decisions. Although these small nuclear RNAs (snRNAs), termed U1, U2, U4, U5, and U6 snRNA, are present in equal stoichiometry within the spliceosome, we found that their relative levels vary by an order of magnitude during development, across tissues, and across cancer samples. Physiologically relevant perturbation of individual snRNAs drove widespread gene-specific differences in alternative splicing but not transcriptome-wide splicing failure. Genes that were particularly sensitive to variations in snRNA abundance in a breast cancer cell line model were likewise preferentially misspliced within a clinically diverse cohort of invasive breast ductal carcinomas. As aberrant mRNA splicing is prevalent in many cancers, we propose that a full understanding of such dysregulated pre-mRNA processing requires study of snRNAs, as well as protein splicing factors. Together, our data show that the RNA components of the spliceosome are not merely basal factors, as has long been assumed. Instead, these noncoding RNAs constitute a previously uncharacterized layer of regulation of alternative splicing, and contribute to the establishment of global splicing programs in both healthy and malignant cells.

Alternative pre-mRNA splicing, which permits the expression of multiple transcript isoforms from a single gene, affects almost all multiexon human genes (Wang et al. 2008). Alternative splicing plays correspondingly crucial roles during normal biological processes such as development and cell type specification (Graveley 2001; Pan et al. 2008; Chen and Manley 2010; Kalsotra and Cooper 2011; Ellis et al. 2012; Yang et al. 2016). Conversely, dysregulation of alternative splicing characterizes many genetic diseases and cancers (Dvinge and Bradley 2015; Dvinge et al. 2016; Scotti and Swanson 2016; Climente-González et al. 2017) and is sufficient to drive disease initiation, progression, and therapeutic response (David and Manley 2010; Papaemmanuil et al. 2011; Yoshida et al. 2011; Graubert et al. 2012; Imielinski et al. 2012; Quesada et al. 2012; Martin et al. 2013; Zhang and Manley 2013). Accordingly, substantial effort has been devoted to identifying and characterizing the factors that control alternative splicing programs in both healthy and diseased cells.

Factors involved in the splicing process can be roughly categorized as “basal” or “regulatory,” depending upon whether or not they are required for all splicing. In this simplified view, basal factors are required to catalyze the splicing process itself, whereas regulatory factors promote or repress splicing. Canonical basal factors include those that constitute the spliceosome, whose core components are the NineTeen Complex (NTC) and five small nuclear ribonucleoprotein complexes (snRNPs, pronounced “snurps”), known as U1, U2, U4, U5, and U6 (Fig. 1A). Canonical regulatory factors, on the other hand, may not be part of the core spliceosome itself. Instead, these regulatory factors typically bind specific enhancer or silencer sequences in pre-mRNA to promote or repress splicing (Fu and Ares 2014; Gerstberger et al. 2014). Canonical basal factors are ubiquitously expressed in all cells because they are required for splicing to occur, whereas cell-type–specific expression of regulatory factors contributes to the establishment and maintenance of distinct splicing programs in different cells. Dysregulated expression or mutations affecting specific splicing factors can alter the normal splicing program to drive genetic, dysplastic, and neoplastic disease (Raj and Blencowe 2015; Dvinge et al. 2016).

**Figure 1.**
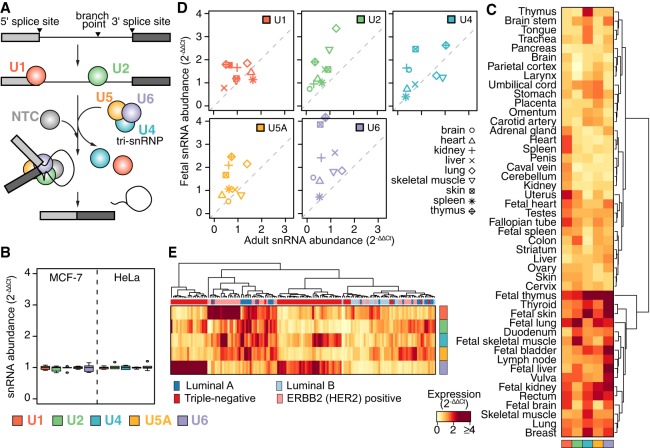
Spliceosomal snRNA abundance is highly variable. (*A*) Simplified schematic of a single round of splicing, showing individual steps: recognition of the 5′ and 3′ splice sites by the small nuclear ribonucleoprotein complexes (snRNPs) containing the U1 and U2 snRNAs, respectively; recruitment of the U4/U6.U5 tri-snRNP; exit of the U1 and U4 snRNAs and rearrangements of the snRNPs into the conformation required for the active spliceosome; excision of the intron lariat; and ligation of the two adjacent exons. Red indicates U1; green, U2; blue, U4; orange, U5; purple, U6. (*B*) Reproducibility of ΔCt values from our microfluidic real-time quantitative PCR-based assay to measure snRNA levels across five biological and three technical replicates, using the MCF-7 and HeLa cell lines. For the calculation of ΔCt, the mean of the 7SK RNA, the signal recognition particle RNA (7SL) and 5S rRNA within each tissue was used as a reference. ΔΔCt was calculated relative to the median across individual snRNA within each cell line. (*C*) Heatmap of relative snRNA abundance across 47 healthy tissues, represented as 2^−ΔΔCt^. ΔCt values calculated as in *B*. ΔΔCt values are relative to the median values across all tissues. (*D*) Expression level (2^−ΔΔCt^) of snRNAs in adult versus fetal samples from identical tissues. Colors as in *A*. For comparison with the technical and biological variability (*B*), scale of the *y*-axis is kept identical. (*E*) Variations in snRNA abundance across 144 primary breast cancer specimens, calculated as in *C*. The column color bar indicates the intrinsic breast cancer subtypes, as defined by immunohistochemistry (IHC) on these samples.

Although it is attractive to label molecules involved in splicing as being purely basal or purely regulatory, recent studies have shown that for some factors, this categorization is an oversimplification that does not accurately reflect their biological role. Core components of the spliceosome can play regulatory, in addition to basal, roles. Even if a particular core spliceosomal protein is required for splicing to occur, developmental stage–specific or tissue-specific variation in its expression level can confer a regulatory role on the protein. For example, the core spliceosomal protein SmB/B′ regulates splicing of a cassette exon within its own pre-mRNA, as well as hundreds of other cassette exons (Saltzman et al. 2011). Other core spliceosomal proteins participate in regulation of alternative splicing (Park et al. 2004; Pacheco et al. 2006; Perez-Santángelo et al. 2014; Wickramasinghe et al. 2015) and display tissue- or development-specific expression patterns (Grosso et al. 2008). Recent systematic screens for alternative splicing regulators have likewise uncovered regulatory potential for multiple components of the core splicing machinery (Papasaikas et al. 2015; Han et al. 2017). Similarly, spliceosomal proteins can be subject to recurrent somatic mutations or aberrant expression in many cancers (Dvinge et al. 2016), and even perturbation of snRNP biogenesis has been implicated in oncogenesis (Koh et al. 2015). Breast cancer likewise displays subtype-specific dependencies on the abundance of distinct components of the spliceosome (Hsu et al. 2016; Chan et al. 2018). These studies suggest that the repertoire of splicing factors that play regulatory roles may be substantially larger than is currently realized and that such factors play important roles in both healthy and malignant cells.

Because core protein components of the spliceosome can act as regulatory factors, we wondered whether the RNA components of the spliceosome might similarly contribute to splicing regulation. Each of the five snRNPs contains a cognate U-rich small nuclear RNA (snRNA). The U1 and U2 snRNAs are responsible for recognizing the 5′ splice site and branchpoint upstream of the 3′ splice site, followed by the U4/U6.U5 tri-snRNP joining the spliceosome before the rearrangements that ultimately lead to the U6 snRNA catalyzing the actual splicing reaction (Fica et al. 2015). These snRNAs are present in constant stoichiometry within the spliceosome and are strictly required for splicing to occur. snRNAs therefore seem like canonical basal factors whose depletion would simply lead to global reductions in splicing efficiency. Instead, however, several disease-associated perturbations in snRNA levels give rise to cell-type–specific changes in splicing that preferentially affect specific genes (Zhang et al. 2008; Jia et al. 2012; Ishihara et al. 2013).

By analogy with snRNA perturbation in disease, endogenous variation in snRNA levels could potentially enable these RNAs to regulate alternative splicing. However, it is unknown whether such variation occurs in healthy cells. Only 100–200 nt in length, snRNAs are not detected by most large-scale assays routinely used in functional genomics (e.g., microarrays and most RNA-seq protocols), unless those assays are specifically designed to target short nonpolyadenylated RNA species. snRNAs levels could potentially vary during development, between cell types, or in healthy versus cancerous cells, but their levels have never been systematically quantified across those biological axes. Here, we systematically tested the hypothesis that endogenous variation in snRNA levels confers regulatory capacity on these RNAs by quantifying snRNA expression across tissue types, developmental stages, and disease states. We then ectopically perturbed snRNA levels to investigate how snRNA abundance shapes alternative splicing across the human transcriptome.

## Results

### Absolute and relative snRNA abundance show extreme variation

We first tested whether snRNA levels were relatively constant, as might be expected from their equal stoichiometry within the spliceosome itself, or whether, instead, snRNA levels were variable, as is common for regulatory splicing factors. We used a microfluidic platform to develop a high-throughput quantitative real-time PCR assay to measure levels of all five snRNAs. As U5 has five distinct sequence variants (U5A, U5B, U5D, U5E, and U5F), we focused on the most abundant form, U5A (Krol et al. 1981; Sontheimer and Steitz 1992). We confirmed the robustness and reproducibility of our microfluidic assay by measuring snRNA levels across five biological and three technical replicates from two distinct cell lines (MCF-7 and HeLa), with average Ct values of 10.38 ± 0.18 across replicates (Fig. 1B). We validated the specificity of all primers via amplicon sequencing (Supplemental Fig. S1).

We used our high-throughput assay to systematically measure snRNA abundance across three distinct biological axes for which splicing is known to play critical roles: between tissues, during development, and in healthy versus cancerous cells. We quantified snRNA levels across diverse tissues derived from healthy donors, including 37 adult tissues and 10 fetal tissues, as well as across a cohort of 144 primary breast cancer specimens. This revealed an unexpected degree of variability in both absolute expression levels and relative expression levels of each snRNA across all three biological axes. Within a given tissue, different snRNAs were expressed at levels varying by up to eightfold with respect to each other; conversely, each snRNA showed a similar degree of expression variability across different tissues (Fig. 1C). U1 was present in excess of other snRNAs, as expected from previous studies (Baserga and Steitz 1993). None of the snRNAs showed coordinated expression levels, including U4, U5A, and U6, even though they play intertwined roles as components of the U4/U6.U5 tri-snRNP.

We next compared snRNAs levels between the nine tissues for which both fetal and adult samples were available. Relative levels of U1 were very similar between fetal and adult tissues, whereas the remaining snRNAs, in particular U2 and U6, were almost exclusively expressed more highly in fetal relative to adult samples (Fig. 1D).

Finally, we compared relative snRNA abundance across a cohort of 144 invasive breast cancer samples. Biopsies were selected to represent all breast cancer subtypes (Sorlie et al. 2001), with a focus on the aggressive triple-negative tumors (*N* = 66 triple-negative; 22 Luminal A; 22 Luminal B; 34 ERBB2 [also known as HER2] positive). Even though all samples were taken from breast ductal carcinoma, they showed a similar degree of variability in relative snRNA levels as we observed across our entire panel of human tissues. This variability in snRNAs levels was not random. An unsupervised cluster analysis of the cohort, based solely upon snRNA levels, revealed that most samples showed subtype-specific patterns of snRNA expression (Fig. 1E). The triple-negative samples clustered into two distinct groups with different patterns of relative snRNA expression, perhaps reflecting the well-known heterogeneity of this subtype (Lehmann et al. 2011). We conclude that snRNA levels are extremely variable across a wide range of biological conditions.

### Physiological perturbation of snRNA levels modulates alternative splicing

We next sought to test whether the high physiological variability in snRNA levels that we observed might contribute to the establishment of global splicing programs. Because the abundance of individual snRNAs was not coordinately regulated across tissues or cancer biopsies, we hypothesized that perturbing the expression of a specific snRNA within physiological ranges would modulate splicing. Short nuclear noncoding RNAs are not amenable to RNAi (Ploner et al. 2009), and most snRNAs are present in the genome as multicopy genes, rendering genetic knockouts infeasible. We therefore transiently transfected cells with chemically modified antisense oligos (ASOs) to trigger RNase H–mediated degradation of each specific snRNA, a strategy that has proven effective for targeting U1 snRNA (Liang et al. 2011; Vickers et al. 2011). We focused our knockdown (KD) studies on U1, U2, U4 and U6, but not on U5 because its distinct sequence variants make it resistant to efficient targeting by our ASO strategy.

**Figure 2.**
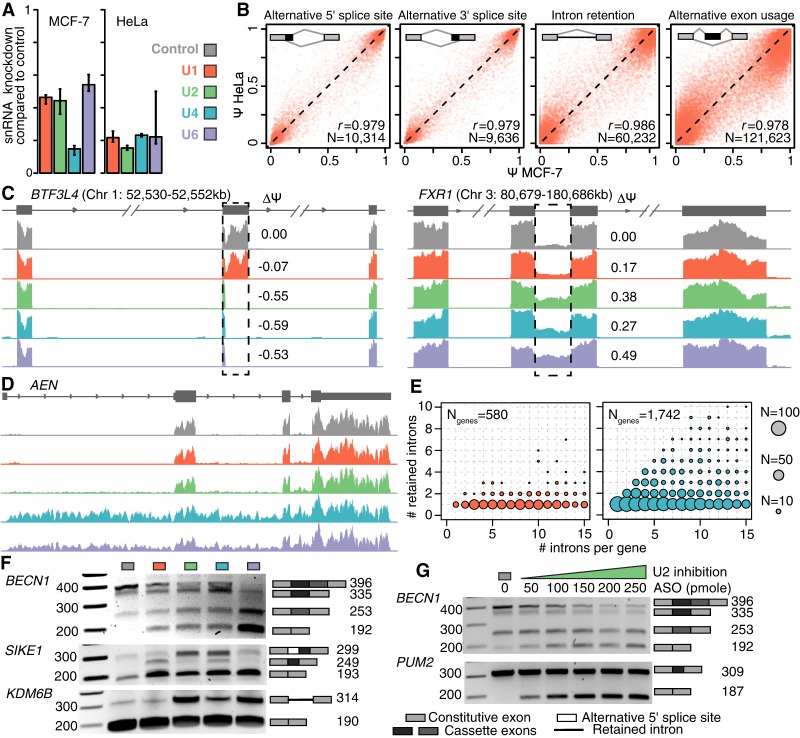
Physiological depletion of snRNAs modulates alternative splicing. (*A*) Levels of each snRNA following knockdown (KD) in MCF-7 and HeLa cells. Levels are relative to cells transfected with a scrambled control oligo. Error bars, 95% confidence intervals, calculated using the balanced repeated replication technique across three technical replicates of each measurement. Red indicates U1; green, U2; blue, U4; purple, U6. (*B*) Alternative splicing induced by U1 snRNA KD in MCF-7 versus HeLa cells. Each dot illustrates an individual splicing event (alternative 5′ splice sites, alternative 3′ splice sites, exons, and introns), represented as percent-spliced-in (PSI; Ψ) values. Plot restricted to events with at least 20 informative reads. (*C*) Alternative splicing of an exon within basic transcription factor 3–like 4 (*BTF3L4*, *left*) and an intron within FMR1 autosomal homolog 1 (*FXR1*; *right*). Dashed boxes indicate the differentially spliced regions. ΔΨ is defined as the fraction of transcripts containing the alternatively spliced region in snRNA KD versus control KD samples. (*D*) Intron retention across an entire transcript of apoptosis enhancing nuclease (*AEN*) following U4 or U6 snRNA KD. (*E*) Number of introns per gene (*x*-axis; plot restricted to genes with 15 or fewer introns) versus introns with a statistically significant increase in intron retention (*y*-axis) following KD of U1 (*left*) or U4 (*right*) snRNA. Circle size is proportional to the number of genes for each (*x*,*y*) combination. (*F*) PCR of select splicing events: multiple adjacent cassette exons (beclin 1, *BECN1*), a combination of a cassette exon and alternative 5′ splice site (suppressor of IKBKE 1, *SIKE1*), and intron retention (lysine demethylase 6B, *KDM6B*). The ladder is included on the *left*, and the *right*-hand side of each gel indicates the splice variants and their predicted sizes. (*G*) Dosage-dependent differential splicing (*BECN1* and pumilio RNA binding family member, *PUM2*) following U2 KD with increasing concentration of antisense oligo (ASO).

We depleted each of U1, U2, U4, and U6 snRNAs in MCF-7 and HeLa cells to levels that were comparable to the variation observed across healthy tissues and our breast cancer cohort (Fig. 2A; Supplemental Fig. S2A). We induced this modest level of depletion, rather than depleting each snRNA to the lowest possible level, in order to mimic physiological variability in snRNA abundance. Although such depletion may not directly reflect a corresponding decrease in snRNP complexes available for assembly into functional spliceosomes on the pre-mRNA, it serves as an in vitro model of the consequences of the observed in vivo variation in snRNA expression. We performed matched RNA-seq following depletion of each snRNA or transfection with a nontargeting control oligo.

We quantified genome-wide alternative splicing of competing 5′ and 3′ splice sites, retained introns, and cassette exons, removal of constitutive introns, and alternative splicing of constitutive splice junctions using two distinct methods. First, we computed isoform usage, defined as percent-spliced-in (PSI or Ψ) values, in each sample for alternative 5′ splice sites, alternative 3′ splice sites, retained introns, and cassette exon events with a probabilistic framework for quantification of splicing across a mixture of isoforms (MISO) (Supplemental Table S1; Katz et al. 2010). Second, we confirmed the robustness of MISO's usage estimates by performing an orthogonal and statistically stringent analysis that used only RNA-seq reads that spanned splice junctions that uniquely defined individual isoforms. (We considered reads spanning exon–intron boundaries as well for the purposes of analyzing intron retention.) We observed high concordance in isoform estimates obtained with these two orthogonal methods, with Pearson's correlations ranging from 0.7–0.9 (Supplemental Fig. S2C). As MISO is more sensitive (because it uses reads within exons and introns as well as junction-spanning reads), we relied on its estimates for our primary analysis. However, as MISO only analyzes differential splicing for predefined isoforms within the annotation database, we augmented our analysis by additionally analyzing all exons and introns for which we could compute Ψ values with reads spanning junctions or exon–intron boundaries (Supplemental Table S2).

We observed concordant patterns of transcriptome-wide alternative splicing in MCF-7 and HeLa cells following KD of each snRNA, with the individual categories of splicing events each showing Pearson's correlations >0.96 between the two cell lines for each snRNA (Fig. 2B; Supplemental Fig. S2B). Given the highly concordant nature of these changes in splicing, we focused subsequent analyses on data from MCF-7 cells. This cell line was established from a pleural effusion of a breast adenocarcinoma patient, and it is therefore particularly biologically relevant to our breast cancer cohort.

Having established our model system, we next tested whether snRNA KD resulted in globally inefficient or failed splicing (suggestive of a purely basal role for snRNAs) or instead preferentially affected specific splice sites, exons, or introns (suggestive of a potential regulatory role for snRNAs). We did not observe classic hallmarks of globally inefficient splicing, such as widespread intron retention. Instead, most splicing changes affected single exons in an snRNA-dependent manner, whereas adjacent upstream and downstream exons were recognized with apparently normal efficiency (Fig. 2C, left). We did observe intron retention, suggestive of failure to recognize splice sites or catalyze splicing; however, typically only single introns were affected, whereas neighboring introns within the same transcript were spliced efficiently (Fig. 2C, right). We did not observe consistent transcriptome-wide differences in gene body read coverage for any snRNA KD relative to control KD (Supplemental Fig. S2D).

Although most genes contained only a single exon or intron that was affected by snRNA KD, a small subset of genes was highly sensitive to perturbation of snRNA levels. For genes such as *AEN*, we observed increased intron retention for most or all introns within the gene following U4 or U6 KD (Fig. 2D). We quantified this effect genome-wide by enumerating the number of retained introns relative to the total number of introns for each gene. For U1 KD, almost all affected genes contained just one retained intron, regardless of the gene structure (Fig. 2E, left). For U4 KD, in contrast, many genes contained multiple retained introns, and a small number of genes showed complete intron retention (Fig. 2E, right). The consequences of U2 and U6 KD were similar to those observed for U1 and U4 KD, respectively (Supplemental Fig. S2E). We validated splicing changes via qRT-PCR across a panel of 14 genes that showed “simple” differential cassette exon usage and intron retention, as well as more complex splicing changes in which multiple adjacent exons and introns displayed differential splicing following snRNA KD (Fig. 2F; Supplemental Fig. S3A).

We tested whether differential splicing induced by snRNA KD showed dose-dependent behavior by transfecting snRNA-targeting ASOs at different concentrations. We transfected cells with varying amounts of the U2 snRNA-targeting ASO and assayed differential splicing in eight splicing events within six genes via qRT-PCR. The most pronounced splicing changes occurred when cells were transfected with the highest concentration of ASO (250 pmol), as expected (Fig. 2G; Supplemental Fig. S3B). However, we consistently observed the same, although quantitatively more modest, splicing changes upon treatment with lower concentrations, suggesting that even modest variation in cellular snRNA abundance impacts alternative splicing. All of the eight distinct splicing events tested (five exons and three introns) showed monotonic behavior as a function of ASO concentrations, consistent with an snRNA-dependent shift in the balance between two or more isoforms per region of the mRNA transcripts queried by PCR. Individual splicing events differed in their susceptibility to modulation of snRNA abundance. For example, the splicing of two adjacent cassette exons in *BECN1* changed in a dose-dependent manner across the entire range of concentrations, whereas exclusion of a cassette exon within *PUM2* reached maximal levels upon treatment with only a modest concentration of U2 snRNA-targeting ASO (Fig. 2G).

We next measured the time-dependent nature of splicing changes in the same six genes following ASO transfection. We observed a gradual change in mRNA species for the first 24 h following treatment, consistent with the transcriptome representing a balance between pre-existing and newly transcribed and spliced mRNA. Our chosen time point (48 h), which likely represents primarily newly transcribed and processed mRNA given that typical mRNAs have half-lives of 5–10 h (Sharova et al. 2009; Tani et al. 2012), showed the largest splicing changes (Supplemental Fig. S3C). It is possible that particular mRNAs are unusually long-lived, such that the abundance of that mRNA at the 48-h time point represents a mixture of pre-existing and newly transcribed and spliced mRNAs. However, in that case, then our results provide an underestimate, rather than overestimate, of the degree of differential splicing caused by snRNA KD.

### Different snRNAs have consistent roles in constitutive and alternative splicing

**Figure 3.**
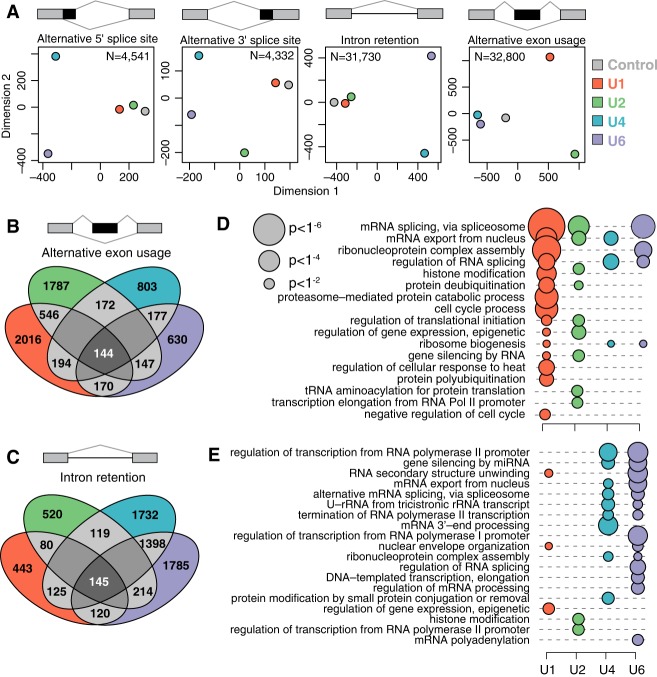
The functions of individual snRNAs are similar in basal and alternative splicing. (*A*) Two-dimensional clustering (multidimensional scaling) of samples based on transcriptome-wide splicing of different categories of splicing events, as in Figure 2B. The clustering was based on the events with the largest degree of variation across samples, as indicated by N. Red indicates U1; green, U2; blue, U4; purple, U6. (*B*,*C*) Overlap between exons (*B*) and introns (*C*) that are differentially spliced following KD of each snRNA in MCF-7 cells. Most events are unique to depletion of individual snRNA. (*D*) Enrichment of Gene Ontology (GO) biological process categories for genes containing differentially spliced exons (*D*) or introns (*E*). The displayed terms were selected based on their combined significance across the four samples. Only terms with at least two ancestors are illustrated. Circle size is proportional to the statistical significance.

As each snRNA has a distinct and well-characterized role in splicing, we wondered whether the observed differential splicing following KD of each snRNA was consistent with the stage of the splicing process when each snRNP joins the spliceosome (Fig. 1A). We clustered control, U1, U2, U4, and U6 KD samples according to how each affected alternative splicing of competing 5′ and 3′ splice sites, intron retention, and alternative exon usage (Fig. 3A). U1 and U2 KD primarily impacted splice site recognition and cassette exon inclusion, consistent with their respective roles in binding the 5′ splice site and branchpoint upstream of the 3′ splice site (Fig. 3A,B). U4 and U6 snRNAs, in contrast, primarily affected intron retention, consistent with their roles in splicing catalysis following splice site recognition (Fig. 3A,C).

### snRNA KD has distinct but convergent biological consequences

We next tested whether the splicing changes that we observed were specific to particular snRNAs or whether, instead, perturbation of any snRNA preferentially affected the same transcripts. Approximately 27% and 29% of differentially spliced cassette exons were shared between U1 and U2 KD, whereas 52% and 50% of differentially retained introns were shared between U4 and U6 KD. This overlap is likely owing to the related roles of U1 and U2 snRNA in defining splice sites and U4 and U6 KD in later stages of spliceosome formation and subsequent splicing catalysis. Nonetheless, perturbation of each snRNA induced a largely distinct program of alternative splicing (Fig. 3B,C). We conclude that a small subset of the transcriptome is sensitive to perturbation of any snRNA but that most transcripts respond to inhibition of only a single snRNA, at least within physiological ranges of snRNA KD.

Because each snRNA was associated with both shared and distinct differential splicing following KD, we wondered whether the same would hold true for the downstream biological pathways affected by those splicing changes. We used Gene Ontology analysis to identify pathways that were enriched for genes containing differentially spliced exons or retained introns, as those were the predominant classes of differential splicing. Many snRNA-modulated exons were located within genes encoding proteins involved in RNA processing, including splicing as well as mRNA transport (Fig. 3D). Protein metabolism was also affected by differential splicing, through regulation of translation as well as protein stability in the form of ubiquitination. Retained introns were enriched within transcripts involved in post-transcriptional control of RNA processing or localization, but transcription itself was also overrepresented (Fig. 3E). The enrichment of snRNA-modulated exons and introns within genes encoding RNA processing factors may reflect that such genes typically contain multiple cassette exons and retained introns, potentially rendering them inherently susceptible to perturbations such as snRNA depletion that induce global differences in alternative splicing. Many splicing factors also engage in auto-regulation by controlling the splicing of their own pre-mRNAs (Ni et al. 2007; McGlincy and Smith 2008; Lareau et al. 2010), which may augment the consequences of snRNA depletion for splicing factor genes.

snRNA-mediated differential splicing affected each of the highlighted biological processes by altering transcripts expressed by multiple genes within each pathway. For example, for mRNA export from the nucleus, altered exon inclusion affected multiple components of the nuclear pore complex (e.g., *NUP160*, *NUP188*, *NUP210*, *NUP85*, *NUP98*), as well as the TREX (transcription-export) complex (e.g., *CHTOP*, *NFX1*, *THOC2*, *THOC6*). Protein stability was affected via differential splicing of cassette exons within genes encoding F-box proteins (e.g., *FBXO22*, *FBXO31*, *FBXO4*, *FBXO44*, *FBXO7*, *FBXO9*), as well as genes encoding E2 ubiquitin-conjugating enzymes (e.g., *UBE2B*, *UBE2G1*, *UBE2G2*, *UBE2N*, *UBE2Q2*, *UBE2R2*).

### snRNA-mediated alternative splicing is affected by mechanisms acting in *cis* as well as in *trans*

**Figure 4.**
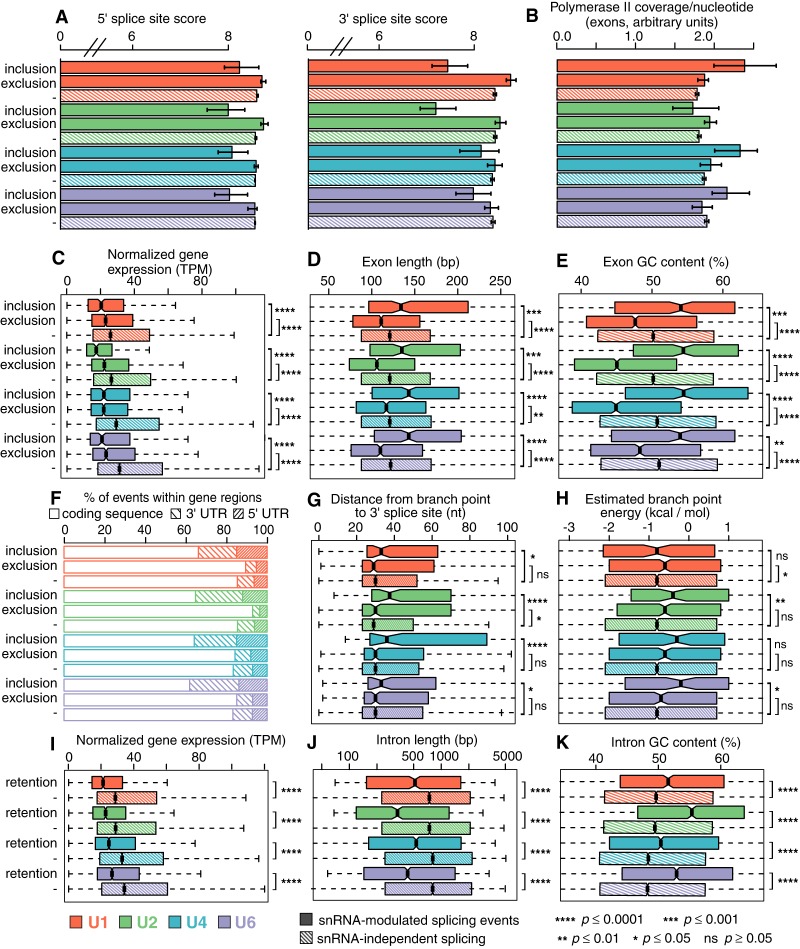
Susceptibility to snRNA depletion is associated with both *cis*- and *trans*-acting features. (*A*) Median scores of 5′ (*left*) and 3′ (*right*) splice sites, calculated with the MaxEnt method for the nucleotide sequences spanning the exon–intron boundaries (Yeo and Burge 2004), for exons with increased inclusion or exclusion following snRNA depletion. “−” indicates the genomic average (exons that are not differentially spliced but that can be reliably detected within the illustrated sample). Error bars, 95% confidence intervals, estimated by bootstrapping. Red indicates U1; green, U2; blue, U4; purple, U6. (*B*) The average per-nucleotide RNA Pol II occupancy of alternatively spliced cassette exons. Error bars, 95% confidence intervals, estimated by bootstrapping. (*C*) Expression of genes containing differentially spliced exons. (TPM) Transcripts per million. Hinges represent the first and third quartile, and notches illustrate the median ±1.58 interquartile range scaled by the number of introns (IQR/√*n*), which approximately corresponds to the 95% confidence interval. Statistical significance is calculated using a two-sided two-sample Wilcoxon test: (****) *P* ≤ 0.0001; (***) *P* ≤ 0.001; (**) *P* ≤ 0.01; (*) *P* ≤ 0.05; (ns) *P* ≥ 0.05. Distribution of lengths (*D*) and average GC content (*E*) of exons showing differential inclusion. Statistical significance as in C. (*F*) Location of differentially spliced exons with the 5′ UTR, coding sequence, or 3′ UTR of their gene. Distance between branchpoints and 3′ splice sites (*G*) and the estimated energy for U2 snRNA/branchpoint base-pairing (*H*) across all exons for which one or more branchpoints present within 200 nt upstream of the 5′ end on the exon (i.e., a 3′ splice site) could be identified from currently annotated branchpoints (Pineda and Bradley 2018). Statistical significance as in *C*. Outliers are excluded, but the full range of branchpoint distances and energies is displayed in Supplemental Figure S6, A and B. (*I*) Expression of genes containing differentially spliced introns. TPM and statistical significance as in *C*. “−” indicates the genomic average (introns that are not differentially retained but that can be reliably detected within the illustrated sample). As human introns are more variable in their lengths than exons, the full range of intron lengths and GC content is displayed in Supplemental Figure S6, E and F. Distribution of lengths (*J*) and average GC content (*K*) of introns that are retained following snRNA KD.

We next sought to determine why some, but not most, splice sites were preferentially sensitive to variable snRNA levels. As snRNA KD depletes levels of factors that are required for splice site recognition or splicing catalysis, we hypothesized that affected and unaffected splice sites might be particularly “weak” or “strong,” respectively. We therefore measured the approximate strength of each 5′ and 3′ splice site by comparing it to the genome-wide consensus for those splice sites using the MaxEnt method (Yeo and Burge 2004). Contrary to our expectations, cassette exons that were preferentially included following KD of any snRNA had weaker 5′ and 3′ splice sites than the genomic average, whereas cassette exons that were preferentially excluded following U1 or U2 KD had modestly stronger 5′ and 3′ splice sites (Fig. 4A). We did not observe any systematic differences in sequence motifs at 5′ or 3′ splice sites (Supplemental Fig. S4).

We next tested whether factors other than the splice site themselves might contribute to alternative exon usage following snRNA KD. As transcriptional rate has been previously shown to influence cassette exon recognition (De La Mata et al. 2003; Fong et al. 2014), we wondered whether fast or slow transcription might similarly render specific exons sensitive or resistant to snRNA KD. We used RNA polymerase (Pol) II occupancy (Honkela et al. 2015) as an indicator of transcriptional rates: Given constant levels of gene expression, reduced transcriptional speed indicates higher Pol II occupancy and vice versa. Cassette exons showing increased inclusion following U1, U4, or U6 KD were characterized by increased Pol II occupancy relative to the genomic average, as well as relative to cassette exons that were preferentially skipped following depletion of those snRNAs (Fig. 4B). Although Pol II occupancy is an imperfect proxy for transcriptional speed, these results suggest that slower transcription of specific exons facilitates their recognition under conditions of lower snRNA abundance.

Increased Pol II occupancy of exons whose inclusion were promoted by snRNA KD could, in principle, arise from increased gene expression (e.g., high density of fast-moving Pol II molecules) rather than slower transcriptional rates. We therefore tested for a relationship between gene expression and responsiveness to snRNA KD. Increased gene expression did not explain the increased Pol II occupancy of exons that were promoted by snRNA KD. Instead, these exons were preferentially located within genes showing lower expression than genes containing exons in which snRNA KD promoted exon skipping (Fig. 4C). Genes containing exons that responded to snRNA KD, whether with increased inclusion or exclusion, tended to be expressed at lower levels than the genomic average. Included exons were typically longer than excluded exons for all four snRNAs (median of 134–146 bp versus 106–117 bp across snRNAs) (Fig. 4D) and had a higher GC content (median of 54% versus 46% GC) (Fig. 4E). To investigate whether this difference in nucleotide composition reflected the presence or absence of particular binding sites for RNA-binding proteins, we performed an ab initio motif enrichment analysis across all differentially spliced exons (Supplemental Fig. S5A). We recovered the expected signal for GC-rich versus AT-rich sequences but did not find other enriched or depleted sequence motifs that might be bound by known splicing enhancers or repressors. Exons that showed increased inclusion upon snRNA KD were enriched within both the 5′ and 3′ UTRs relative to the coding sequence of their parent genes (Fig. 4F).

Most human introns contain multiple branchpoints that may be used by the splicing machinery (Mercer et al. 2015; Pineda and Bradley 2018). We therefore tested whether intron or exons whose splicing was sensitive to snRNA KD showed unusual branchpoint positions relative to the 3′ splice site or particularly weak or strong complementarity to the U2 snRNA. We first determined the distance between branchpoints and the downstream 3′ splice site for all exons with at least one annotated branchpoint. The average number of branchpoints located upstream of the differentially spliced exons did not differ, and there was little to no difference in relative branchpoint position between all exons and those excluded upon snRNA depletion. However, for exons whose inclusion was promoted by snRNA KD, we observed a significant shift toward more distal branchpoints for all snRNAs (Fig. 4G; Supplemental Fig. S6A). Such exons also had branchpoints that showed poorer complementarity to the U2 snRNA, a trend that reached statistical significance for U2 and U6 snRNA KD (Fig. 4H; Supplemental Fig. S6B). The effect was most pronounced for U2 snRNA KD, consistent with U2 snRNA's role in base-pairing with the branchpoint region.

In contrast to cassette exons, introns whose recognition was affected by snRNA KD had neither weaker nor stronger 5′ and 3′ splice sites than the genomic average, suggesting that the initial recognition of exon/intron boundaries was not a determining factor for subsequent intron removal versus retention. Likewise, no systematic differences were observed for their branchpoints, neither in terms of distance to 3′ splice site or in predicted U2 snRNA/branchpoint binding strength (Supplemental Fig. S6C,D). Introns that were retained following snRNA KD were preferentially located within lowly expressed genes (Fig. 4I). Differentially retained introns tended to be short and GC rich. Introns that were retained following snRNA KD had a median length of 469 bp versus 773 bp for introns that were not sensitive to snRNA KD (Fig. 4J; Supplemental Fig. S6E). KD-responsive introns were ∼1.9%–5.9% more GC rich than were unresponsive introns (Fig. 4K; Supplemental Fig. S6F). Those trends are consistent with previous observations that intron length and GC content are strongly associated with an increased propensity toward intron retention (Dvinge and Bradley 2015). We next tested whether specific sequence motifs were enriched or depleted in KD-responsive introns, but did not detect any signals beyond the difference in GC content (Supplemental Fig. S5B) that we also observed for exons. Unlike for exons, we did not observe enrichment for retained introns in UTRs versus coding sequence (Supplemental Fig. S6G).

### Breast cancer splicing profiles overlap with snRNA-modulated events

**Figure 5.**
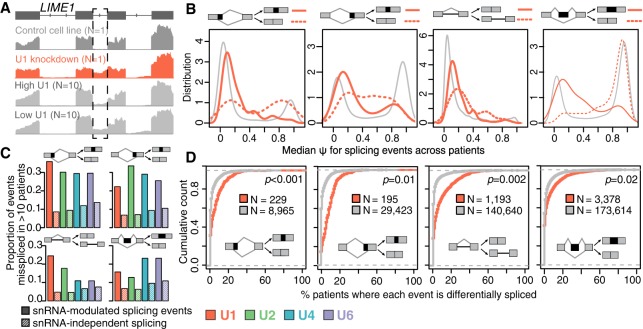
Dysregulated splicing in breast cancer is enriched for snRNA-responsive splice sites and exons. (*A*) snRNA-responsive intron excision within Lck interacting transmembrane adaptor 1 (*LIME1*). Dashed box indicates differentially retained intron, for which U1 snRNA depletion promotes intron retention. (*Top* rows) RNA-seq read coverage from control and U1-depleted MCF-7 cells. (*Bottom* rows) Read coverage from the 10 breast cancer biopsies with the highest and lowest U1 levels. (*B*) Distribution of Ψ values across breast cancer specimens (*N* = 136), stratified by U1 snRNA KD-sensitive (red) and -insensitive (gray) splicing in MCF-7 cells. Dashed lines indicate splicing events for which the alternative spliced sequence is preferentially excluded from the mature transcript following U1 snRNA KD in MCF-7 cells. Solid lines indicate events for which the alternatively spliced sequence is preferentially included following U1 snRNA KD. For retained introns, the dashed line indicates intron retention. Plots are restricted to exons and introns that were annotated as alternatively spliced (see Methods). Note that events that are uniformly excluded or included from the mature mRNAs across the cohort have Ψ values close to zero and one, respectively, whereas intermediate Ψ values indicate heterogeneous splicing that varies within or across patients. (*C*) Proportions of snRNA-dependent and snRNA-independent events that are differentially spliced in at least 10 primary breast cancer biopsies relative to patient-matched peritumoral normal samples (*N* = 107). Samples are from the TCGA breast cancer cohort. Red indicates U1; green, U2; blue, U4; purple, U6. (*D*) Cumulative density functions comparing differential splicing of U1 snRNA KD-sensitive (red) and -insensitive (gray) splicing events across breast cancer patients, as in *C*. The plots illustrate the percentage of primary breast cancer biopsies showing aberrant splicing relative to their patient-matched normal sample, across the TCGA cohort. Splicing events were stratified as U1 snRNA KD-sensitive (red) and -insensitive (gray) based upon the observed splicing in MCF-7 cells. (N) Number of events that could be reliably quantified in the U1 snRNA KD sample. Statistical significance was calculated using the Kolmogorov–Smirnov test.

We next tested whether variable snRNA expression might contribute to the splicing dysregulation that characterizes most cancers. We observed a similar magnitude of variability in snRNA expression within our breast cancer cohort as we achieved via snRNA KD in our MCF-7 model (Fig. 1E). We therefore hypothesized that the exons and introns that were differentially spliced following snRNA depletion in vitro (in MCF-7 cells) would be preferentially misspliced in vivo (in breast cancer samples). To test this hypothesis, we performed deep RNA-seq for 136 of the invasive ductal carcinomas in our cohort and quantified global patterns of splicing. Anecdotal inspection of specific events, such as a retained intron within *LIME1*, revealed that biopsies with particularly low or high levels of a given snRNA frequently showed splicing patterns mimicking those observed following KD of that snRNA in MCF-7 cells (Fig. 5A).

The biopsies that we characterized came from a genetically and clinically heterogeneous cohort, from which we intentionally selected samples representing multiple subtypes of cancer. Many factors in addition to snRNA levels influence the gene expression and splicing programs in each cancer; for example, different breast cancer subtypes show statistically significant different levels of intron retention relative to peritumoral normal tissue (Dvinge and Bradley 2015). We therefore next tested whether snRNA-sensitive splicing events were frequently subject to consistent splicing dysregulation by measuring the uniformity of splicing for individual events across our entire cohort. For exons that were not affected by snRNA depletion, we observed a typical “on/off” splicing pattern, in which the major isoform in almost all patients corresponded to near-complete inclusion or exclusion of the exon (Fig. 5B, left, exemplified by U1). In contrast, snRNA-responsive exons showed variable exon inclusion across the cohort, consistent with frequent alternative splicing/missplicing. We observed a similar pattern for retained introns, as well as alternative 5′ and 3′ splice site events that responded to snRNA KD (Fig. 5B; Supplemental Fig. S7A). We conclude that splice sites and exons that are particularly responsive to snRNA levels show unusually variable recognition in different breast cancer patients, consistent with splicing dysregulation.

We next asked whether this unusually variable recognition of snRNA-responsive splice sites and exons corresponded to missplicing, in the sense of generating dysregulation relative to noncancerous tissue. As matched normal control tissue was not available for our breast cancer cohort, we turned to data from The Cancer Genome Atlas (TCGA), for which patient-matched breast cancer and peritumoral normal samples were available. We profiled genome-wide patterns of alternative splicing in cancer samples relative to patient-matched normal tissue and categorized splicing events by their response to snRNA depletion in our in vitro model. The proportion of snRNA-modulated versus snRNA-insensitive splicing events varied substantially across patients, as expected for a genetically and clinically heterogeneous cohort (Supplemental Fig. S7B). Nonetheless, the snRNA-modulated events were consistently enriched among events that showed differential splicing between patient-matched cancer and peritumoral normal samples. We defined frequently misspliced events as those which differed between cancer and matched normal samples for 10 or more patients. Such frequently misspliced events showed strong and statistically significant enrichment for overlap with snRNA-modulated events relative to snRNA-independent events that we identified in vitro (Fig. 5C; Supplemental Fig. S7B). For example, competing 5′ and 3′ splice sites, retained introns, and cassette exons that were frequently misspliced in primary breast cancer showed enrichments for U1 KD-responsive events with associated *P*-values of 0.001, 0.01, 0.002, and 0.02, respectively (Fig. 5D). Splicing events that were frequently misspliced in primary tumors relative to matched normal tissues showed highly similar and significant enrichments for most U2, U4, and U6 KD-responsive events, although the effect was less pronounced for retained introns (Supplemental Fig. S7C).

## Discussion

Our data indicate that snRNAs play an unexpectedly complex role in establishing global splicing programs in addition to their well-characterized roles in basal splicing catalysis. As perturbation of snRNA levels with physiological ranges induced widespread differences in alternative splicing, we suggest that snRNAs should be considered to act as regulatory in addition to basal factors.

In this paper, we focused on showing that variable snRNA levels influence splice site and exon recognition, particularly in the context of breast cancer. However, our characterization of snRNA levels across multiple biological axes (Fig. 1) clearly shows that snRNA levels are equally variable between tissues and developmental time points as they are between breast cancer patients. It is likely that snRNA levels play important and as-yet-unrecognized roles in establishing tissue-specific and developmental stage–specific splicing programs.

It is challenging to attribute snRNA-mediated changes in splicing to a single underlying mechanism for a number of reasons. The copy number of snRNA transcripts within the cell may not be directly proportional to the number of functional snRNP complexes that assemble on the pre-mRNA to form the active spliceosome. snRNPs may also possess splicing-independent functions, as has been shown for depletion of the U1 snRNA and snRNP, which results in premature cleavage and polyadenylation of a subset of mRNA substrates (Kaida et al. 2010). Other snRNAs may likewise impact RNA maturation through orthogonal, as-of-yet unknown cellular roles. Although we identified both *cis*- and *trans*-associated features that correlated with responsiveness to snRNA KD, the fundamental mechanisms underlying snRNA-mediated changes in splicing remain unknown. None of the features that we identified were strongly predictive of the observed splicing changes. Further work is required to determine why some splice sites and exons are so sensitive to altered snRNA abundance, whereas most splice sites and exons are resistant to perturbation of snRNA levels within physiological ranges.

Although the stoichiometry of both snRNAs and proteins within the spliceosome is well established (Will and Lührmann 2011; Zhang et al. 2017), there is nevertheless evidence that depleting the cellular abundance of individual spliceosomal proteins modulates individual splice site usage (Park et al. 2004; Pacheco et al. 2006; Perez-Santángelo et al. 2014; Wickramasinghe et al. 2015). This may happen through kinetic mechanisms, such as preferential recruitment of rate-limiting snRNPs to strong compared with weak splice sites (Wickramasinghe et al. 2015). We hypothesize that similar mechanisms explain how variability in snRNA levels influences alternative splicing. If a particular snRNP is recruited to two competing splice sites at different rates, then a reduction in the corresponding snRNA could therefore result in altered recognition or processing of one splice site relative to its competing counterpart, resulting in differential production of specific mRNA isoforms. Because we observed differential splicing following snRNA KD that was consistent with the known roles of each snRNA in basal splicing catalysis (Fig. 3), we speculate that each snRNA affects alternative splicing through distinct mechanisms. Although KD of each snRNA resulted in a unique splicing profile, U1 and U2 KD preferentially impacted exon inclusion, whereas U4 and U6 KD preferentially impacted intron retention. These tendencies are consistent with the early requirement for U1 and U2 snRNPs’ interactions with the pre-mRNA. In contrast, as U4 and U6 snRNAs base pair with one another in the U4/U6.U5 tri-snRNP before spliceosome formation on the pre-mRNA, alterations in the relative abundance of those snRNAs might impede or promote the assembly of the precatalytic spliceosomal B complex and subsequent splicing catalysis.

As our data indicate that snRNA dysregulation shapes the global transcriptome of breast cancer, we speculate that snRNA dysregulation may contribute to tumorigenesis itself. Multiple prior studies have identified connections between snRNAs and cancer. For example, overexpression of U1 snRNA may promote procancer gene expression (Cheng et al. 2017), and U2 snRNA fragments are potential blood-based biomarkers for multiple cancers (Kuhlmann et al. 2014, 2015; Baraniskin et al. 2016; Köhler et al. 2016). We here observed a clear association between snRNA levels and the intrinsic breast cancer subtypes (Sorlie et al. 2001). The ERBB2 (HER2) subtype displayed high concurrent levels of U1 and U5A, whereas the two clusters of triple-negative samples showed higher relative abundance of U6 or comparatively low levels of U2 and U5A. Further work is required to determine whether these differences in relative snRNA abundance are downstream from key regulators of each subtype, such as the estrogen or progesterone receptors, or instead contribute to the establishment of the gene expression programs that define each subtype. Regardless, our data show that contrary to their traditional classification as “basal” components of the splicing machinery, spliceosomal snRNA constitute a previously uncharacterized layer of regulation of transcriptome-wide alternative splicing. A full understanding of the regulatory machinery that establishes splicing programs in both benign and malignant cells therefore requires study of not just protein splicing factors but also noncoding RNA components of the splicing machinery.

## Methods

### Primary breast cancer sample collection and processing

Primary breast specimens were collected by the Fred Hutchinson Cancer Research Center/University of Washington Breast Specimen Repository with approval of the local IRB. Women were diagnosed with invasive breast carcinoma between 2002 and 2015 and had no prior cancer diagnosis and no neoadjuvant treatment (radiation, chemotherapy, or hormone) before tissue collection. Breast tissue taken as core biopsies or surgical specimens were flash-frozen and embedded in OCT. Each biopsy was macrodissected into 10–20 10-µm sections to enrich for tumor cells. Total RNA and DNA were isolated concurrently across sections from each biopsy, using the AllPrep DNA/RNA/miRNA universal kit (Qiagen) according to the manufacturer's recommended protocol. Immunohistochemistry (IHC) results were taken from medical records. Additional testing for a marker of proliferation MKI67 (also known as Ki-67 [MIB-1, Dako]) was performed by IHC. Tumor subtypes were determined using IHC results for estrogen receptor alpha (ER, encoded by *ESR1*), progesterone receptor (PR, encoded by *PGR*), erb-b2 receptor tyrosine kinase 2 (ERBB2; also known as human epidermal growth factor receptor 2, HER2), and MKI67. The subtypes were defined as follows using the commonly accepted names: Luminal A (ER and/or PR positive, ERBB2 negative, MKI67 < 14%), Luminal B (ER and/or PR positive, ERBB2 negative, MKI67 ≥ 14%), HER2 (ERBB2 positive, any ER/PR/MKI67), and triple-negative (ER, PR, and ERBB2 all negative, any MKI67).

### Tissue specimens from healthy individuals

Total RNA from adult and fetal human tissues was obtained from Agilent Technologies. Adult tissue samples originate from individual donors, with the exception of breast, cerebellum, larynx, liver, lung, spleen, stomach, and trachea, which were from a pool of two to six donors each. Both genders were represented, with the age ranging from 56 ± 16.5 yr (median ± SD). All fetal samples were from donors aged 18–23 wk. Brain, lung, and skeletal muscle were from individual donors, and the remaining fetal samples were pooled across two to 17 donors, aged 18–23 wk.

### Cell culture and snRNA KD

MCF-7 breast cancer cells (from American Type Culture Collection; HTB22) and HeLa cervical cancer cells (gift from J Cooper) were maintained in DMEM (4.5 g/L glucose and glutamine, Life Technologies) with 10% fetal bovine serum, and 1% penicillin/streptomycin. MCF-7 cell media were supplemented with 10 µg/mL human recombinant insulin (Life Technologies). snRNA KD was performed using chemically modified RNA–DNA hybrids (Integrated DNA Technologies), with a nontargeting scrambled sequence as control (Supplemental Table S3). Transfection was performed in six-well plates using Lipofectamine RNAiMAX (Invitrogen) following the manufacturer's cell line–specific protocols (MCF-7 reverse transfection; HeLa forward transfection), with 200 pmol ASO and 10 µL RNAiMAX reagent per well in a six-well plate. Cells were harvested after 48 h. Total RNA was extracted using NucleoSpin miRNA (Macherey-Nagel) to collect the small and large RNA fractions combined. For the U2, KD series was performed using the same experimental conditions, with the amount of oligo ranging from 50–250 pmol per well.

### snRNA quantification

snRNA primers validated to confirm that they produced a single band, and the resulting amplicons were subjected to Sanger sequencing in the forward and reverse location. snRNA levels in MCF-7 and HeLa were quantified with real-time qPCR using the VeriQuest SYBR Green One-Step method (Thermo Fisher Scientific), with 100 pg/µL total RNA and 10 µM primer per reaction (Supplemental Table S3). Samples were processed using the ΔΔCt method, using the 7SK RNA from the *RN7SK* gene as a reference, the KD with scrambled control as a calibrator sample, and the primer-specific amplification efficiency α estimated from a four-sample dilution series. snRNA levels from human tissues and breast cancer specimens were quantified using the Biomark HD 48.48 dynamic array (Fluidigm). Total RNA (5 ng/µL) was mixed with sample loading buffer (Fluidigm), Fast EvaGreen qPCR master mix Lo-ROX (Biotium), reverse transcriptase and RiboSafe RNase inhibitor (both SensiFAST one-step, Bioline). Arrays were primed, loaded, and run according to the instrument specifications. All samples were run in triplicate and were processed using the ΔCt method, using the mean Ct value across 7SK, the signal recognition particle RNA (7SL) from *RN7SL1* and 5S rRNA as a pseudoreference, to correct for variations in amount of RNA input. ΔΔCt values were calculated relative to the median ΔCt values across all tissues and the entire breast specimen cohort, respectively.

### RNA sequencing

RNA-seq libraries for cell lines samples were created with the KAPA Stranded mRNA-seq kit (Kapa Biosystems) with poly(A) selection. Invasive breast carcinomas with a sufficient amount of high-quality total RNA available were processed with KAPA Stranded RNA-seq kits with RiboErase for ribosomal RNA depletion, owing to potential mRNA fragmentation during storage of the specimens. Both types of libraries were prepared following the manufacturer's instructions, with 500 ng of total RNA as input, RNA fragmentation for 7 min at 94°C, and 10 PCR cycles of cDNA amplification. Library quality was assessed using the Agilent 2200 TapeStation. Barcoded RNA-seq libraries were sequenced in triplex on the Illumina HiSeq 2500 using single-ended 67-bp reads.

### Genome annotations

Alternative splicing events were categorized as cassette exons, retained introns, and competing 5′ and 3′ splice sites, according to the MISO v2.0 annotations (Katz et al. 2010). Constitutively spliced junctions were defined as adjacent splice junctions in which alternative splicing was not detected in any isoform of the UCSC knownGene track (Meyer et al. 2013). Separate annotation files were created for RNA transcripts and splice junctions for the read mapping. The RNA transcript annotation is a combination of isoforms present in MISO v2.0 (Katz et al. 2010) plus the UCSC knownGene (Meyer et al. 2013) and the Ensembl 71 gene annotation (Flicek et al. 2013). The annotation for RNA splice junctions contains an enumerating of all possible combinations of annotated splice sites as previously described (Hubert et al. 2013).

### RNA-seq read mapping

The RNA-seq reads were mapped in five stages. (1) Bowtie (Langmead et al. 2009) and RSEM (Li and Dewey 2011) were used to map all reads to the UCSC hg19 (NCBI GRCh37) human genome assembly. Realigning the reads to the GRCh38 human genome assembly would not impact our analysis, as the primary update to the genome assembly was the addition of data related to population variation and filling of gaps, such as centromeric regions (Schneider et al. 2017). Our conclusions regarding alternative splicing would therefore not be significantly affected. Reads were mapped to the gene annotation file using RSEM with the arguments --bowtie-m 100 --bowtie-chunkmbs 500 --calc-ci --output-genome-bam after modifying RSEM version 1.2.4 to call Bowtie version 1.0.0 with the -v 2 mapping strategy. (2) The resulting BAM file was filtered to remove alignments that had a mapq score of zero or where the splice junction overhang was ≤5 bp. (3) Next, all the so-far-unaligned reads were extracted from the BAM file and aligned to the RNA splice junction file using TopHat2 version 2.0.8b (Kim et al. 2013) with the arguments --bowtie1 --read-mismatches 2 --read-edit-dist 2 --no-mixed --no-discordant --min-anchor-length 6 --splice-mismatches 0 --min-intron-length 10 --max-intron-length 1000000 --min-isoform-fraction 0.0 --no-novel-juncs --no-novel-indels --raw-juncs. Other required parameters (--mate-inner-dist and --mate-std-dev) were determined for each sample by mapping reads to constitutive coding exons according to the exon_utils.py script in MISO. (4) The newly aligned reads were filtered again, using the same criteria as in stage 2. (5) Finally results from RSEM and TopHat2 were merged to create a combined BAM file containing all alignments. All transcriptome and splicing alignments were strand-specific.

### Splicing validation

Genes were selected to represent different responses to snRNA depletion, including splicing events displaying only moderate sensitivity to individual or multiple snRNAs. cDNA was generated using the iScript reverse transcription supermix (Bio-Rad) containing a mix of oligo(dT) and random primers, and PCR was performed using *Taq* DNA Pol (Invitrogen), both following the manufacturers’ instructions. PCR primers were designed to span one or more alternative splicing events (Supplemental Table S3).

### Metagene RNA-seq read coverage

The read coverage for each individual nucleotide across all exons annotated as belonging to a protein-coding gene was extracted from the mapped BAM files. Each full-length gene was divided into 50 bins, and the reads per nucleotide were averaged within each bin and divided by the number of million mapped reads per sample. Low-coverage genes were filtered by removing genes in which no bins exceeded a scaled coverage of 0.1. To adjust for variation in gene expression levels, bins were normalized to the median within each gene. A summarized metagene profile was created by calculating the median of each bin across all genes.

### RNA splicing quantification

MISO (Katz et al. 2010) and v2.0 of its annotations were used to quantify splicing, using PSI values for all cassette exons, retained introns, and competing 5′ and 3′ splice sites. Reads directly spanning the splice junctions were used for detection and quantification of alternative splicing of constitutive junctions and retention of constitutive introns, as previously described (Hubert et al. 2013). All subsequent analyses were restricted to splicing events that were alternatively spliced in our data based on at least 20 relevant reads (i.e., reads supporting either or both isoforms). For subsequent analysis of retained introns and alternative exon usage, all statistically significant differentially spliced events were included regardless of whether they were annotated as being alternative or constitutive. For alternative events, the more sensitive PSI values from MISO were used, and for constitutive events, the stringent PSI values derived from junction-spanning reads were used. In total, our analysis incorporated 168,000 introns, 210,000 exons, and approximately 3.3 million potential junctions arising from novel splice site usage. The number of individual splicing events that could be detected using the above-mentioned criteria are summarized in Supplemental Table S2.

### Sample clustering

Hierarchical clustering (heatmaps) of the relative snRNA levels within tissues and breast cancer patients was performed with the “ward.D2” method (Ward 1963) using data that had been standardized according to the ΔΔCt approach. MCF-7 snRNA KD samples were clustered using multidimensional scaling (also known as principal coordinates analysis). The distances were calculated using the “canberra” method, sum(|*x*_*i*_ − *y*_*i*_|/ |*x*_*i*_ + *y*_*i*_|), using only events that were alternatively spliced in at least one sample and had more than 20 reads supporting either of the spliced isoforms.

### Identification of differentially spliced isoforms

Events were defined as differentially spliced between a KD and the control if they satisfied the following criteria: (1) There were at least 20 relevant reads in both samples (reads supporting either or both isoforms), (2) there was a change in isoform ratio of at least 10%, and (3) there was a Bayes factor of statistical significance greater than or equal to one. Wagenmakers's framework (Wagenmakers et al. 2010) was used to compute Bayes factors for differences in splicing of individual events between sample pairs. Splice site motifs for differentially spliced events were calculated by creating position weight matrices of the 4 nt from the −2 to +6 position for 5′ splice sites and −8 to +2 position for 3′ splice sites. Sequence logos were created with the seqLogo package v1.26.0 in Bioconductor (Gentleman et al. 2004). For the analysis of differential splicing versus features in *cis* and *trans*, all statistical comparisons were performed using the nonparametric two-sided two-sample Wilcoxon tests (Mann–Whitney *U* test), as most features were not normally distributed.

### Branchpoint analysis

All branchpoints, genomic coordinates, and estimated U2 snRNA/pre-mRNA branchpoint energies were obtained from Pineda and Bradley (2018). For the initial mapping of branchpoint coordinates, all introns containing at least one identified branchpoint were included. For potentially differentially spliced exons, we extracted all branchpoints in the intron upstream of their 5′ end. Finally, we filtered the results to include only branchpoints within a 200-nt distance from the 3′ splice site.

### Identification of enriched motifs

To identify sequence motifs ab initio, all *k*-mers of length four, five, or six were queried. For exons, the occurrence of each motif was calculated in exons that were included upon snRNA KD and compared with the occurrence in exons that were excluded. For introns, the motif occurrences in differentially retained introns were compared with all nonretained introns. Statistical significance was calculated using the nonparametric Kolmogorov–Smirnov test, and only motifs with a *P*-value <0.01 and a log_2_ enrichment above 1.5 were included.

### Gene Ontology enrichment for differentially spliced events

Enrichment of biological process terms from the Gene Ontology was performed using the R package GOseq (Young et al. 2010) using the “Wallenius” method. Splicing events were mapped back to genes and compared with a background universe consisting of all spliced protein-coding genes with an expression level above one in at least two of the four KD samples, after normalizing the expression level within each sample using the trimmed mean of M values (TMM) method (Robinson and Oshlack 2010) with scaling factors calculated based on all protein-coding genes. The resulting false-discovery rates were corrected using the Benjamini-Hochberg approach. Only terms with at least two ancestors were tested to eliminate parent terms associated with generic biological processes.

### Transcription rates

RNA Pol II ChIP-seq and rRNA-depleted RNA sequencing data for MCF-7 cells were obtained from GSE62789 (Honkela et al. 2015) using only the untreated samples. To obtain gene expression estimates, the RNA-seq data were processed and normalized as described above, with a maximum of one mismatch. The ChIP-seq reads were mapped to the human transcriptome using RSEM, following the same strategy. Genes with an expression less than one TPM in the reanalyzed RNA-seq data from Honkela et al. (2015) were removed from the subsequent ChIP-seq analysis in order to reduce noise. For each individual exon in the remaining genes, the average per-nucleotide Pol II read coverage across the entire exon from start to end position was calculated.

### Analysis of breast cancer specimens from The Cancer Genome Atlas

TCGA RNA-seq data from breast cancer patient-matched tumors and samples from the adjacent normal tissue were obtained and processed as previously described (*N* = 107) (Dvinge and Bradley 2015). To avoid bias owing to events or genes predominantly expressed in vitro or in vivo, splicing events were filtered to include only events with a minimum of 20 event-specific reads, which could be detected in both MCF-7 and at least 20% of patients. For the cumulative density function, the filtered events were stratified as snRNA KD-sensitive or -insensitive based upon the observed splicing in MCF-7 cells. Statistical significance was determined using a one-sided Kolmogorov–Smirnov test, with the same number of steps as the cohort size (*N* = 107).

## Data access

The raw sequencing reads from the cell lines in this study have been submitted to the NCBI Gene Expression Omnibus (GEO; https://www.ncbi.nlm.nih.gov/geo/) under accession number GSE107163.

## Supplementary Material

Supplemental Material
