# Supplementary material for: RNA components of the spliceosome regulate tissue- and cancer-specific alternative splicing
Source: Genome Res. 2019 Oct;29(10):1591–604. doi: 10.1101/gr.246678.118 (PMC6771400; doi:10.1101/gr.246678.118)
Supplement: Supplemental Material [file supp_29_10_1591__index.html]

RNA components of the spliceosome regulate tissue- and cancer-specific alternative splicing — RNA components of the spliceosome regulate tissue- and cancer-specific alternative splicing — Supplemental Material 

# RNA components of the spliceosome regulate tissue- and cancer-specific alternative splicing

## Supplemental Material

- Supplemental\_Fig\_S1.pdf
- Supplemental\_Fig\_S2.pdf
- Supplemental\_Fig\_S3.pdf
- Supplemental\_Fig\_S4.pdf
- Supplemental\_Fig\_S5.pdf
- Supplemental\_Fig\_S6.pdf
- Supplemental\_Fig\_S7.pdf
- Supplemental\_Table\_S1.xlsx
- Supplemental\_Table\_S2.xlsx
- Supplemental\_Table\_S3.xlsx
