# Supplementary material for: RNA components of the spliceosome regulate tissue- and cancer-specific alternative splicing
Source: Genome Res. 2019 Oct;29(10):1591–604. doi: 10.1101/gr.246678.118 (PMC6771400; doi:10.1101/gr.246678.118)
Supplement: Supplemental Material [file supp_gr.246678.118_Supplemental_Fig_S1.pdf]

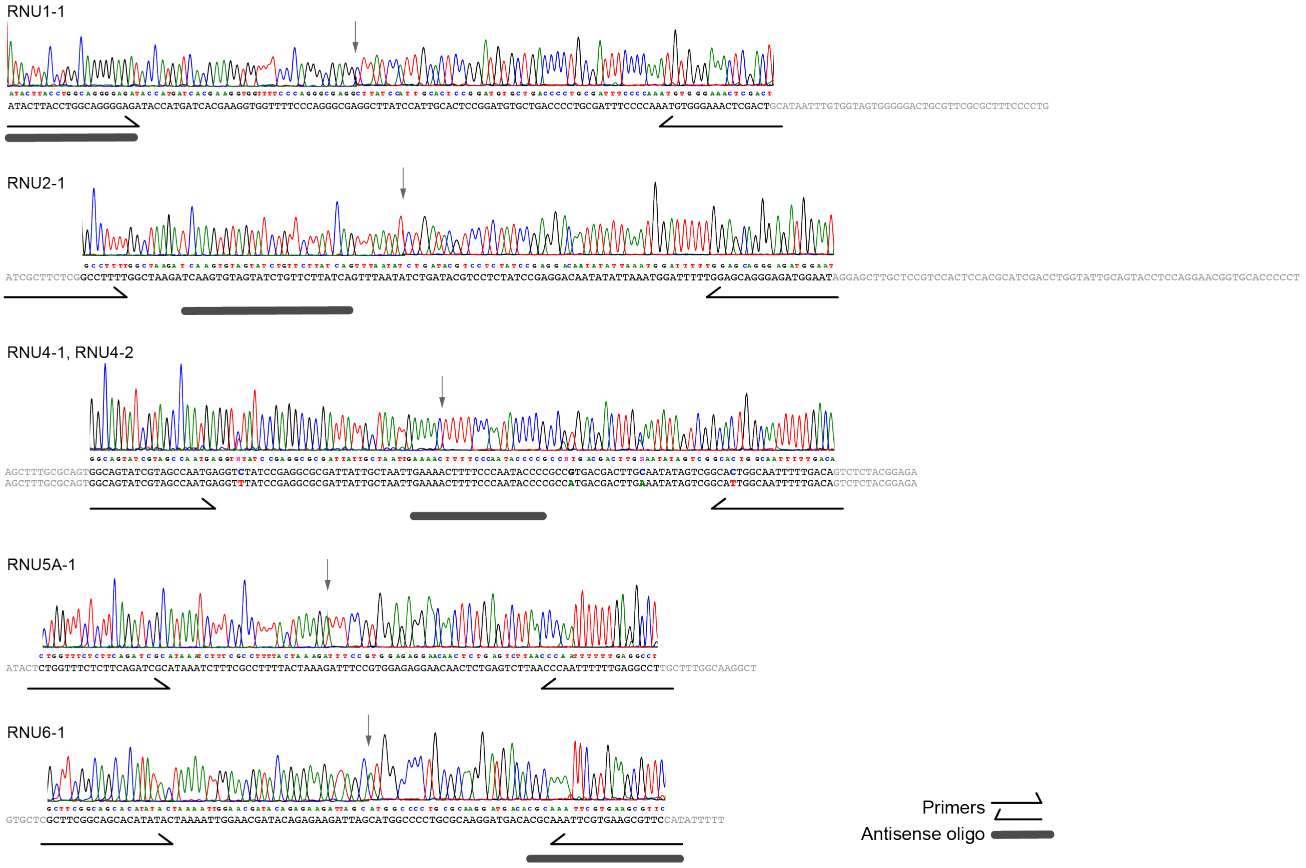

Supplemental Figure S1: Validation of primer specificity and location of antisense oligos for knockdown.

Amplicon sequence trace, gene sequence, location of forward and reverse primer, and location of antisense oligo for each of the snRNAs. For the gene sequence, the region included in the sequence trace is highlighted in black, and the remaining sequence in grey. To encompass their entire sequence, the amplicons were sequenced in both the forward and the reverse orientation. Vertical grey arrows mark where the sequence traces from the two orientations were joined. U4 snRNA is encoded by two almost identical genes, with nucleotide differences indicated in the gene sequences. The gene variants are expressed in approximately equal amounts according to the sequence traces. The antisense oligo is located in a region that is identical in both genes.
