# Supplementary material for: RNA components of the spliceosome regulate tissue- and cancer-specific alternative splicing
Source: Genome Res. 2019 Oct;29(10):1591–604. doi: 10.1101/gr.246678.118 (PMC6771400; doi:10.1101/gr.246678.118)
Supplement: Supplemental Material [file supp_gr.246678.118_Supplemental_Fig_S2.pdf]

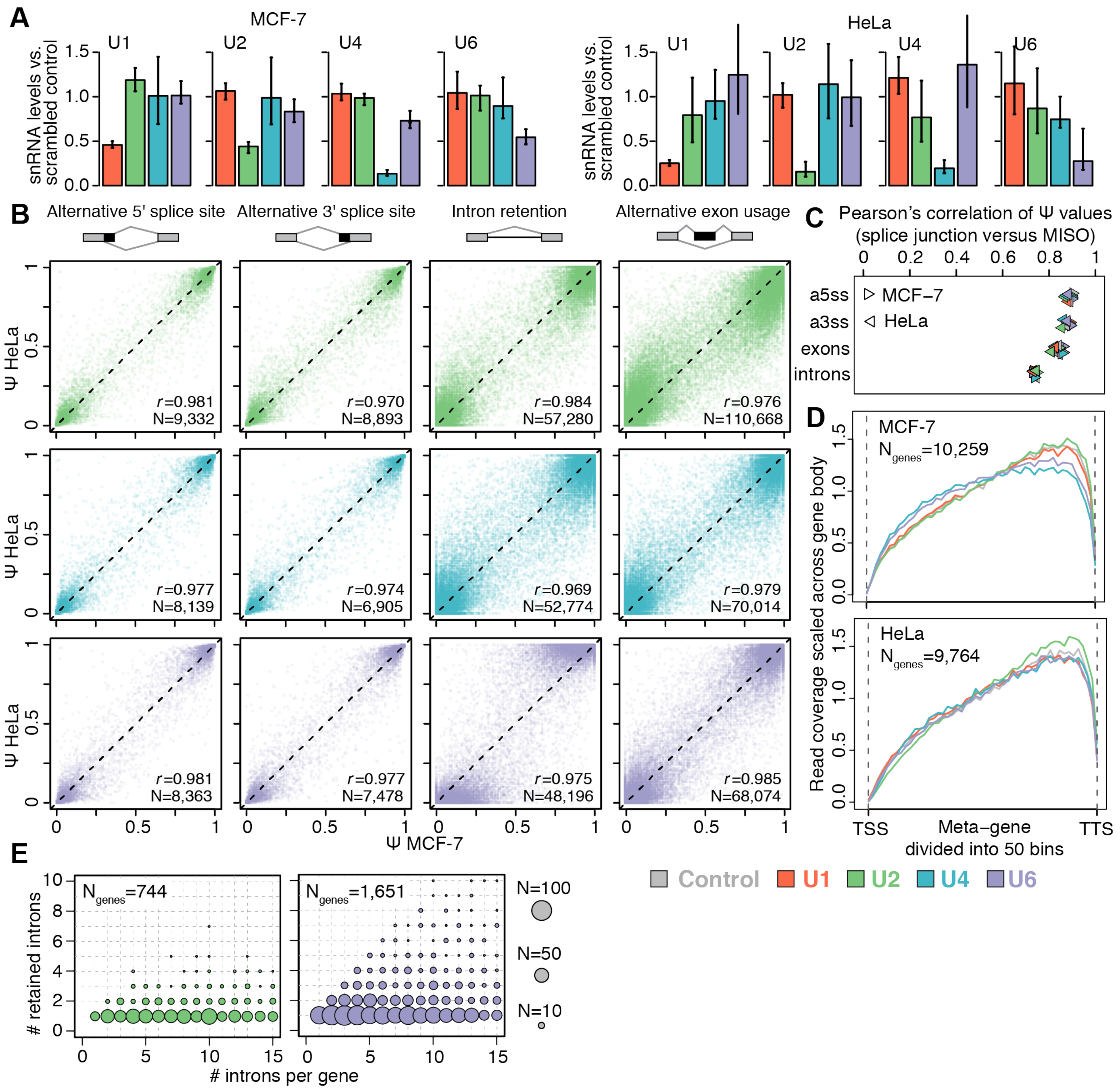

Supplemental Figure S2: Depletion of snRNAs across cell lines and intron retention.

(A) Levels of all snRNAs in samples with knockdown of a single target snRNA in MCF-7 (left) and HeLa (right) cells. Levels are illustrated as $\alpha^{-\Delta\Delta Ct}$, where α is the primer-specific amplification efficiency, estimated using a standard curve, using 7SK as a reference RNA and cells transfected with a scrambled control oligo as a reference sample. Error bars illustrate 95% confidence intervals, calculated using the balanced repeated replication technique across three technical replicates of each measurement. Red=U1, green=U2, blue=U4, purple=U6.

(B) Alternative splicing induced by U1 snRNA KD in MCF-7 versus HeLa cells for U2 (*top*), U4 (*middle*), and U6 (*bottom*). Each dot illustrates an individual splicing event (alternative 5' splice sites, alternative 3' splice sites, exons, and introns), represented as percent spliced in (PSI, Ψ) values. Plot restricted to events with at least 20 informative reads.

(C) Pairwise Pearson's correlation between Ψ values calculated using MISO or junction-spanning reads within each cell line. a5ss; alternative 5' splice sites, a3ss; alternative 3' splice sites.

(D) Read coverage between the transcription start site (TSS) and transcription termination site (TTS) of a meta-gene comprised of all expressed protein-coding genes. For each gene the RNA-seq read coverage is calculated in 50 bins along the gene body. The bias towards the 3' end is consistent with the selection of poly(A)-tailed RNAs during RNA-seq library preparation.

(E) Number of introns per gene (x axis; plot restricted to genes with <= 15 introns) versus introns with a statistically significant increase in intron retention (y axis) following KD of U2 (*left*) or U6 (*right*) snRNA. Circle size is proportional to the number of genes for each (x,y) combination.
