# Supplementary material for: RNA components of the spliceosome regulate tissue- and cancer-specific alternative splicing
Source: Genome Res. 2019 Oct;29(10):1591–604. doi: 10.1101/gr.246678.118 (PMC6771400; doi:10.1101/gr.246678.118)
Supplement: Supplemental Material [file supp_gr.246678.118_Supplemental_Fig_S3.pdf]

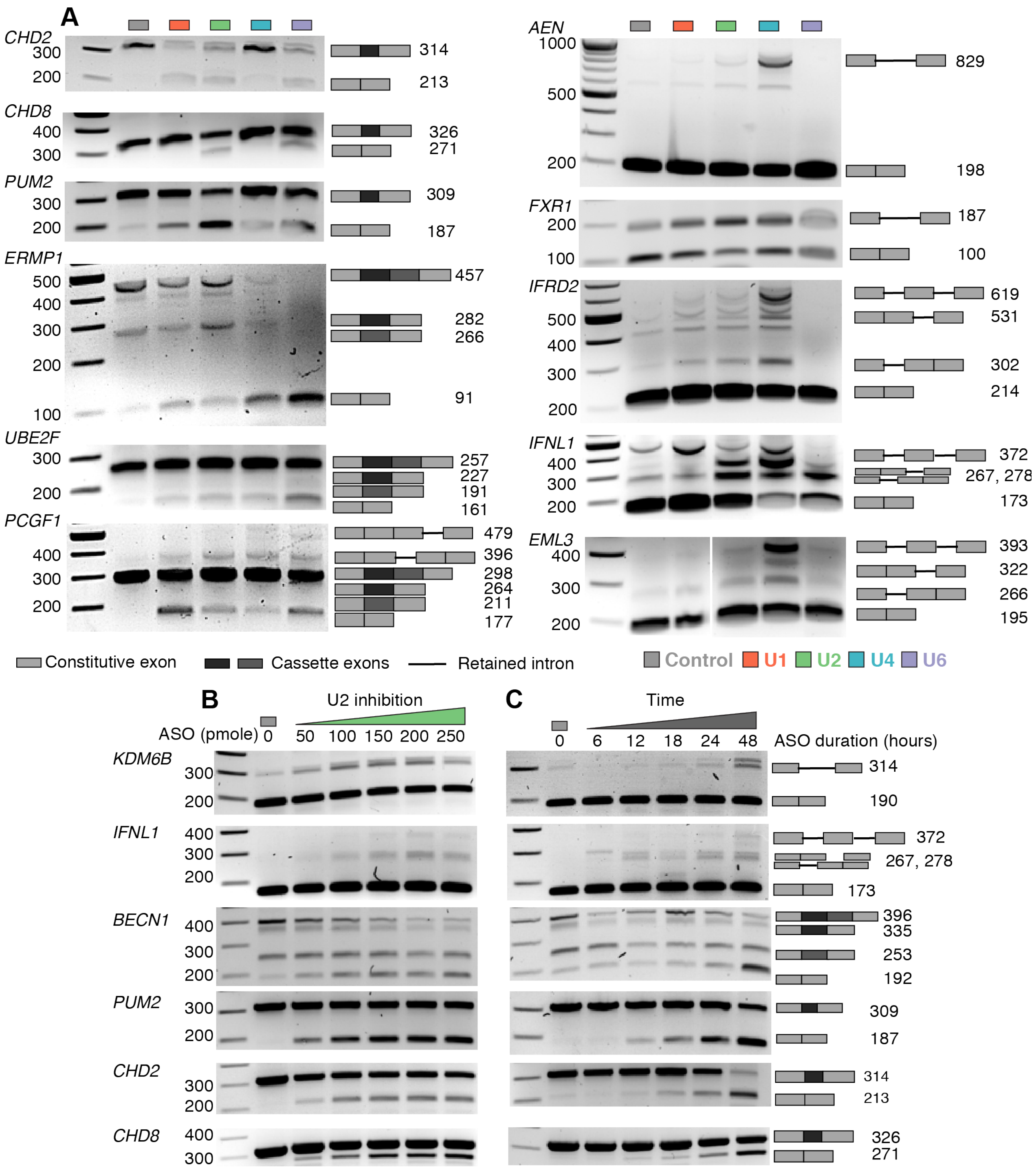

Supplemental Figure S3: PCR validation of alternative splicing.
(A) PCR of select splicing events, using primers spanning cassette exons or retained introns. The right-hand side of each gel indicates the splice variants and their predicted sizes, including for cassette exons that are not observed in any of the samples (*e.g.,* polycomb group ring finger 1, *PCGF1)*. Splicing events were selected to include a range of possible outcomes, such as where alternative splicing was only observed in a subset of snRNA-knockdown samples (*e.g.,* chromodomain helicase DNA binding protein 8, *CHD8,* and EMAP like 3, *EML3*); where it was the degree of differential splicing differed (*e.g.,* ubiquitin conjugating enzyme E2 F, *UBE2F,* and pumilio

RNA binding family member, *PUM2*); or where multiple alternatively spliced exons or introns were observed within the same transcript (*e.g.,* interferon lambda 1, *IFNL1,* and endoplasmic reticulum metallopeptidase 1, *ERMP1*)

(B) Dosage-dependent differential splicing following U2 knockdown with increasing concentration of antisense oligo (ASO), from 0 to 250 pmole.

(C) Time-dependent differential splicing following U2 knockdown, at 0 to 48 hours following 200 pmole ASO treatment.
