# Supplementary material for: RNA components of the spliceosome regulate tissue- and cancer-specific alternative splicing
Source: Genome Res. 2019 Oct;29(10):1591–604. doi: 10.1101/gr.246678.118 (PMC6771400; doi:10.1101/gr.246678.118)
Supplement: Supplemental Material [file supp_gr.246678.118_Supplemental_Fig_S4.pdf]

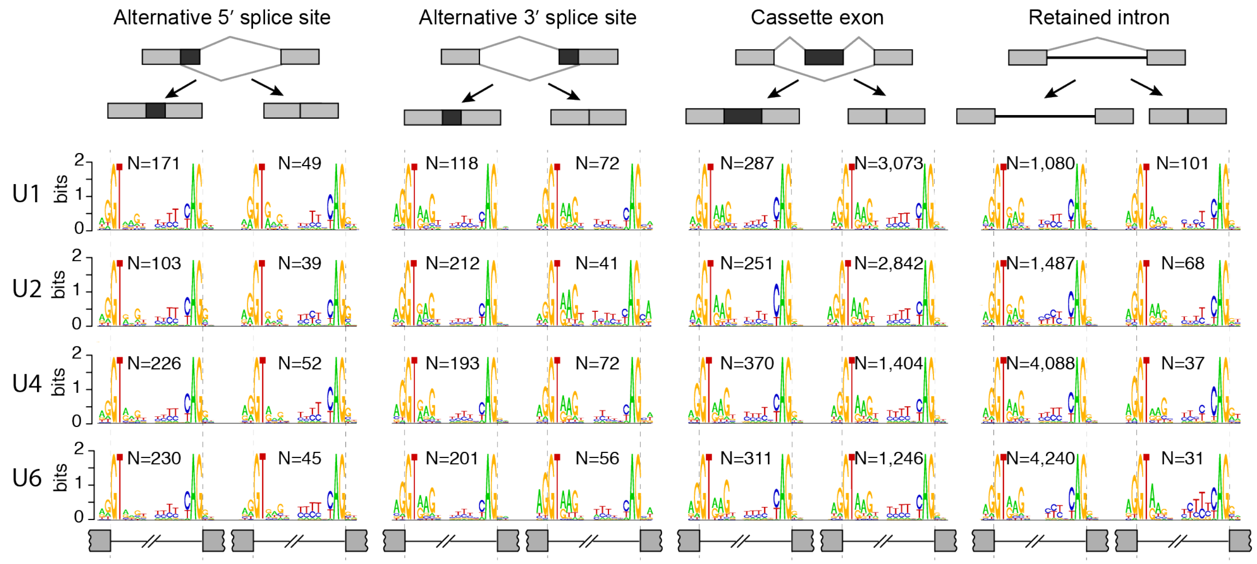

Supplemental Figure S4: Consensus 5' and 3' splice site sequences for all differentially spliced events in the individual snRNA knockdown versus control samples (MCF-7). Nucleotide heights correspond to their frequency across the differentially spliced events (N). Motifs are derived from the DNA gene sequence, using T instead of U. Grey boxes (*bottom*) indicates the location of the upstream and downstream exons, with the intervening intron truncated at the double slashes.
