# Supplementary material for: RNA components of the spliceosome regulate tissue- and cancer-specific alternative splicing
Source: Genome Res. 2019 Oct;29(10):1591–604. doi: 10.1101/gr.246678.118 (PMC6771400; doi:10.1101/gr.246678.118)
Supplement: Supplemental Material [file supp_gr.246678.118_Supplemental_Fig_S5.pdf]

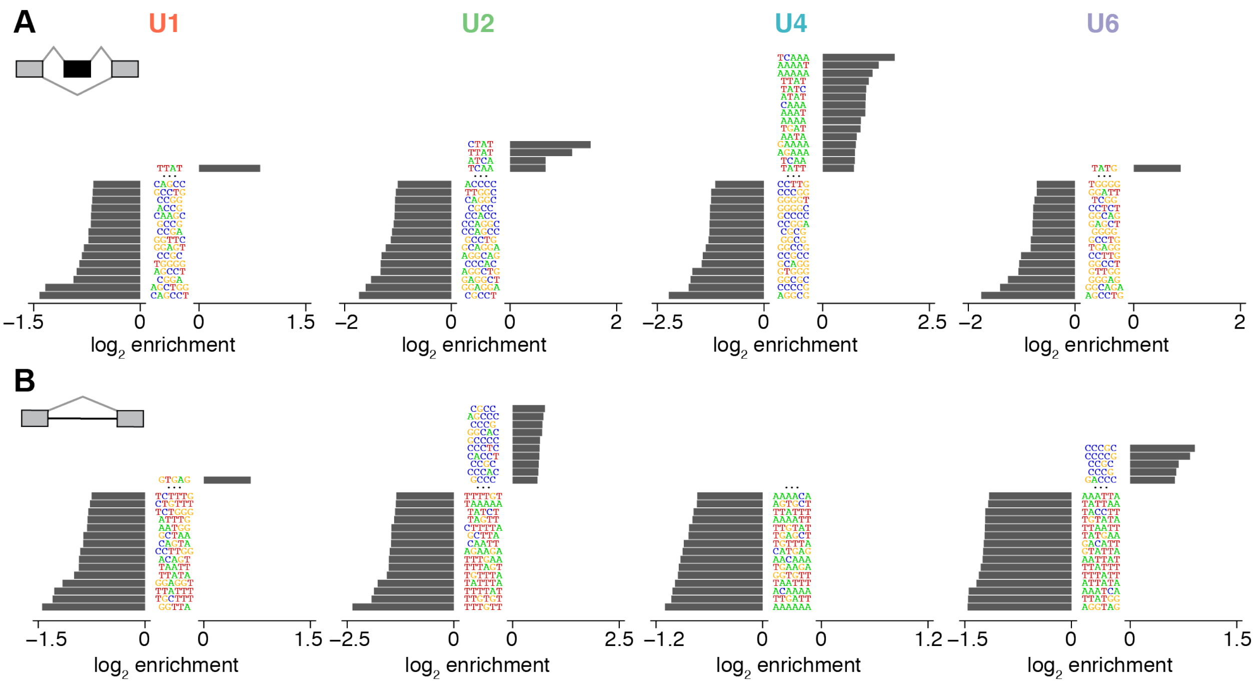

Supplemental Figure S5: Enrichment of sequence motifs in exons and introns affected by snRNA abundance.

Depleted (left) and enriched (right) motifs in *(A)* cassette exons that are differentially included versus excluded upon snRNA depletion and *(B)* introns that are retained versus un-affected introns.
