# Supplementary material for: RNA components of the spliceosome regulate tissue- and cancer-specific alternative splicing
Source: Genome Res. 2019 Oct;29(10):1591–604. doi: 10.1101/gr.246678.118 (PMC6771400; doi:10.1101/gr.246678.118)
Supplement: Supplemental Material [file supp_gr.246678.118_Supplemental_Fig_S7.pdf]

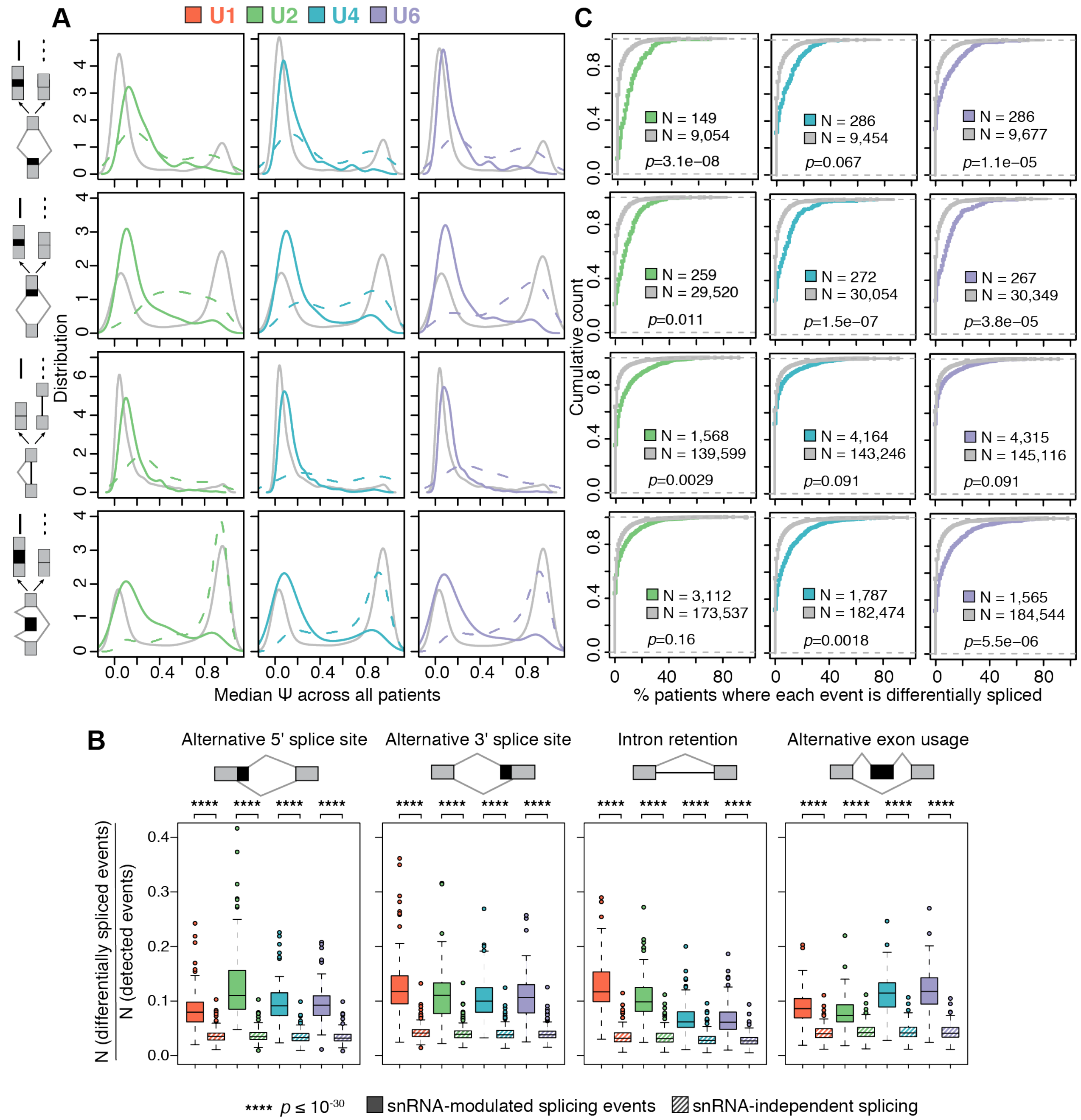

Supplemental Figure S7: snRNA-dependent versus independent splicing events in breast cancers *(A)* Distribution of Ψ values across breast cancer specimens (N = 136), stratified by snRNA KD-sensitive (colored) and -insensitive (grey) splicing in MCF-7 cells. Dashed lines indicate splicing events for which the alternative spliced sequence is preferentially excluded from the mature transcript following snRNA KD. Solid lines indicate events for which the alternatively spliced sequence is preferentially included following snRNA KD. For retained introns, the dashed line indicates intron retention. Plots restricted to exons and introns that were annotated as alternatively spliced (see Methods). Note that events that are uniformly excluded or included from the mature mRNAs across the cohort have Ψ values close to 0 and 1, respectively, while intermediate Ψ values indicate heterogeneous splicing that varies within or across patients. Red=U1, green=U2, blue=U4, purple=U6.

(B) Distribution of snRNA-dependent and -independent events that are differentially spliced in primary breast cancer biopsies relative to patient-matched peritumoral normal samples, relative to the number of events in each category that could be robustly detected within individual patient. Samples are from the TCGA breast cancer cohort (N = 107). Statistical significance was calculated using a paired two-sided t-test.

(C) Cumulative density functions comparing differential splicing of snRNA KD-sensitive (colored) and -insensitive (grey) splicing events across breast cancer patients, as in *(B)*. The plots illustrate the percentage of primary breast cancer biopsies exhibiting aberrant splicing relative to their patient-matched normal sample, across the TCGA cohort. Splicing events were stratified as snRNA KD-sensitive (colored) and -insensitive (grey) based upon the observed splicing in MCF-7 cells. N, number of events that could be reliably quantified in the snRNA KD sample. Statistical significance was calculated using the Kolmogorov-Smirnov test.
