# Supplementary material for: RNA components of the spliceosome regulate tissue- and cancer-specific alternative splicing
Source: Genome Res. 2019 Oct;29(10):1591–604. doi: 10.1101/gr.246678.118 (PMC6771400; doi:10.1101/gr.246678.118)
Supplement: Supplemental Material [file supp_gr.246678.118_Supplemental_Fig_S6.pdf]

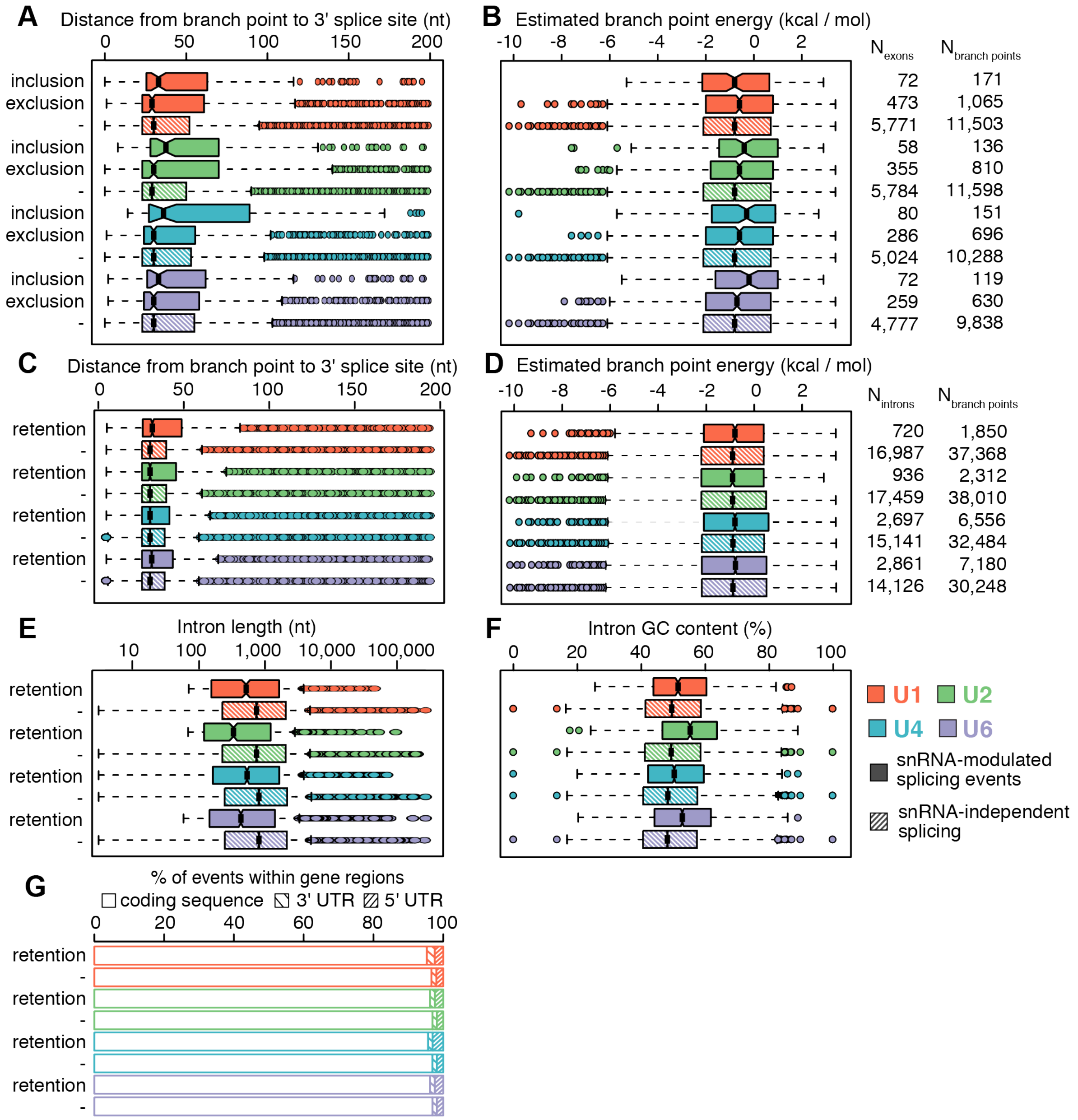

Supplemental Figure S6: Susceptibility to snRNA depletion relative to *cis*- and *trans*-acting features.

(A,C) Distance between branchpoints and 3' splice sites and *(B,D)* the estimated energy for U2 snRNA/branchpoint base pairing, for exons and introns, respectively. 'Exons' ***(A,B)*** depict all differentially spliced exons with increased inclusion or exclusion following snRNA depletion, with '-' indicating the genomic average (exons that are not differentially spliced, but which can be reliably detected within the illustrated sample). 'Introns' ***(C,D)*** depict introns that are retained following snRNA KD, with '-' indicating the genomic average (introns that are not differentially retained, but which can be reliably detected within the illustrated sample). Branchpoint locations and energy were obtained from (Pineda and Bradley 2018). N_{exons} and $N_{introns}$, number of exons or introns for which at least one branch had been identified; $N_{branchpoints}$, the total number of branchpoints located within 200nt of the 3' splice site. *(E)* Distribution of intron lengths and *(F)* GC content, as well as *(G)* location of each intron with the coding sequence or the UTRs of the parent gene. Red=U1, green=U2, blue=U4, purple=U6.
